# A RARE CASE OF ACUTE PULMONARY EMBOLISM MASQUERADING AS ACUTE MYOCARDIAL INFARCTION.

**DOI:** 10.51894/001c.123105

**Published:** 2024-08-30

**Authors:** Karishma A. Doshi

**Affiliations:** 1 Internal Medicine Garden City Hospital

## 87

### INTRODUCTION

Acute pulmonary embolism (APE) presents with varied symptoms, ranging from chest pain to hemodynamic instability. The overlapping symptoms of APE and acute myocardial infarction (AMI) necessitate timely differentiation for appropriate care. Diagnosing APE requires clinicians to maintain a high index of suspicion, and over the years, several scoring tools have been developed to assist with this. This case report details a rare instance where the clinical presentation mimicked myocardial infarction (MI), accompanied by electrocardiogram (ECG) findings indicative of antero-septal MI.

### CASE DESCRIPTION

A 42-year-old male with a medical history of cannabinoid and tobacco dependence, insomnia, and schizophrenia presented with chest pain, dyspnea, and diaphoresis. The patient was hemodynamically stable but tachypneic. Elevated troponin levels and an ECG showing ST elevation in anteroseptal leads prompted immediate transfer to the cath lab. However, no occlusive vessels were found. Subsequently, an elevated D-dimer and RV strain on echocardiogram led to a computed tomographic (CT) angiography for pulmonary embolus (PE), revealing a massive saddle pulmonary embolus. The patient was then returned to the cath lab for thrombectomy.

### DISCUSSION/CONCLUSION

PE has a high mortality rate with a reported 100,000 deaths annually. Key observations and take-home points from this case include: 1) Not all ST elevations with elevated troponins indicate AMI. Maintaining broad differentials and investigating PE is equally imperative in such cases. 2) The estimated mortality rate with intermediate-high risk PE ranges from 3% to 65%. Recent studies highlight an association between time to intervention and morbidity in these patients, emphasizing the importance of reduced door-to-balloon times and a corresponding increase in patients with normal coronaries. 3) A triple-rule-out (TRO) CT scan may serve as an ideal adjunct for evaluating coronary arteries, the aorta, pulmonary arteries, and adjacent intra-thoracic structures simultaneously in patients with acute chest pain.

